# Bioinformatics Strategies in Breast Cancer Research

**DOI:** 10.3390/biom15101409

**Published:** 2025-10-02

**Authors:** Matteo Veneziano, Isabella Savini, Elisa Cortellesi, Valeria Gasperi, Alessandra Gambacurta, Maria Valeria Catani

**Affiliations:** 1 Department of Experimental Medicine, Tor Vergata University of Rome, 00133 Rome, Italy; matteo.veneziano@alumni.uniroma2.eu (M.V.); savini@uniroma2.it (I.S.); elisa.cortellesi@students.uniroma2.eu (E.C.); gasperi@med.uniroma2.it (V.G.); gambacur@uniroma2.it (A.G.); 2 NAST Centre (Nanoscience & Nanotechnology & Innovative Instrumentation), Tor Vergata University of Rome, 00133 Rome, Italy

**Keywords:** breast cancer, biomarker, genomics, transcriptomics, proteomics, metabolomics, drug response

## Abstract

Breast cancer is a heterogeneous disease and a leading cause of cancer-related deaths worldwide, underscoring the urgent need for effective biomarkers to guide diagnosis, prognosis, and therapeutic decisions. Bioinformatics methodologies, including genomics, transcriptomics, proteomics, and metabolomics data analysis, are essential for deciphering the complex molecular landscape of breast cancer. Bioinformatics tools facilitate the identification of differentially expressed genes, non-coding RNAs, and proteins, unraveling crucial pathways involved in tumor initiation, progression, and metastasis. By constructing and analyzing protein–protein interaction networks and signaling pathways, bioinformatics approaches can identify potential diagnostic, prognostic, and predictive biomarkers. Herein, we explore the role of bioinformatics in breast cancer research and its potential application in identifying novel therapeutic targets and predicting drug response, ultimately enabling the development of tailored treatment strategies. We also address the challenges and future directions in utilizing bioinformatics for biomarker discovery and validation, emphasizing the need for robust statistical methods, standardized data analysis pipelines, and collaborative efforts to translate bioinformatics insights into improved clinical outcomes for breast cancer patients.

## 1. Introduction

In 2022, GLOBOCAN estimated 2.3 million new cases of breast cancer (BC) worldwide, making it the most diagnosed tumor and the leading cause of cancer-related deaths among women [1]. BC is an extremely intricate tumor characterized by significant histological and molecular heterogeneity, which influences progression, metastasis formation, and therapy response [2,3]. Consequently, diagnosis and the following appropriate therapeutic plan strictly depend on the integration of histological and molecular characteristics of the tumor, that take into the account grade, stage, lymphovascular invasion, and the presence of axillary lymph node metastasis, but also the status of steroid receptors [estrogen receptor (ER), progesterone receptor (PR)], human epidermal growth factor receptor 2 (HER2), and the Ki67 index (nuclear marker of proliferating cells) [4].

**Figure 1:**
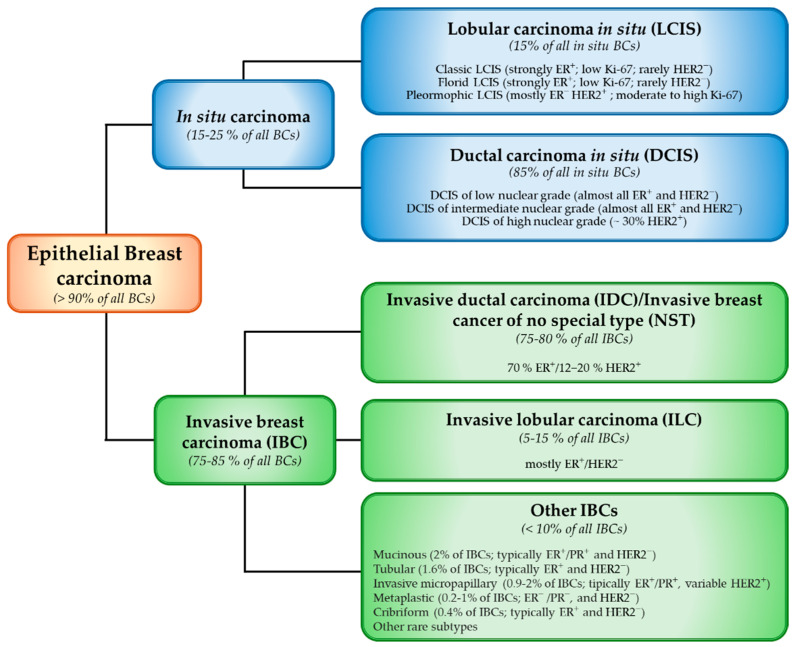
Schematic representation of the current (5th edition) World Health Organization (WHO) classification of epithelial breast carcinomas, including main categories, subtypes, and their relative frequencies. The diagram distinguishes between in situ and invasive carcinomas, further detailing the predominant histological subtypes and their typical receptor status. BC: Breast Cancer; DCIS: Ductal Carcinoma in Situ; ER: Estrogen Receptor; HER2: Human Epidermal Growth Factor Receptor 2; IBC: Invasive Breast Carcinoma; IDC: Invasive Ductal Carcinoma; ILC: Invasive Lobular Carcinoma; LCIS: Lobular Carcinoma in Situ; NST: No Special Type; PR: Progesterone Receptor.

Although BC classification is constantly adapting and evolving, based on the integration of new information from various sources, the standard for diagnosis has been established by World Health Organization (WHO). The primary histologic classification of BC is based on whether the tumor is of epithelial origin, or not. While less than 1% of BCs have non-epithelial origin (e.g., sarcoma deriving myofibroblasts and blood vessel cells), the vast majority of BCs are carcinomas deriving from the epithelial cells of breast lobules and milk ducts [4,5] (Figure 1). This latter group is histologically categorized as in situ carcinoma (further distinguished in lobular and ductal carcinoma, LCIS and DCIS, respectively) and invasive carcinoma (IBC), able to infiltrate the surrounding mammary glandular tissue and metastasize to regional lymph nodes and distant organs. The most common IBC is the invasive carcinoma no special type (NST) [also referred to invasive ductal carcinoma (IDC)], followed by invasive lobular carcinoma (ILC, approximately 10–15% of invasive BC), and other subtypes accounting for less than 10% of BC [4].

On the basis of molecular subtyping [6], BCs are further classified in luminal A-like (ER^+^, PR^+^, HER2^−^, Ki67^low^), luminal B-like (ER^+^ and/or PR^+^, HER2^+/−^, Ki67^low/high^), HER2-enriched (ER^−^, PR^−^, HER2^+^, Ki67^high^) and basal-like [triple-negative breast cancer (TNBC); ER^−^, PR^−^, HER2^−^, Ki67^high^] [3]. BC also exhibits strong familial clustering, with *BRCA1* and *BRCA2* being well-known high-penetrance genes associated with increased risk, and a polygenic origin suggested by medium-low penetrance genes, such as *PALB2*, *PTEN*, *PIK3CA*, *ATM*, *PALB2*, *BARD1*, *CHEK2*, *RAD51C*, *RAD51D*, and *TP53* [7,8,9,10]. Analysis of global gene expression patterns reveals distinct BC subtypes with unique biological behaviors, treatment responses, and prognoses [11,12]. For instance, in DCIS, germline *BRCA1* and *BRCA2* mutations, *HER2* amplification, and negative ER and PR status are observed, reflecting the full spectrum of luminal A, luminal B, HER2-enriched, and basal-like molecular subtypes. Conversely, classic ILCs predominantly express ER and PR and lack *HER2* gene amplification/overexpression. Furthermore, specific etiological risk factors, such as *BRCA1* and *TP53* mutations, are usually associated with TNBC, while *BRCA2* and *PIK3CA* mutations are linked to Luminal A- and B-like BC subtypes [4].

Due to the complex and heterogeneous nature of BC, traditional research methods often fall short in providing a comprehensive understanding of the molecular aberrations within tumors. This is where bioinformatics tools become invaluable, enabling the integration of high-throughput data and clinical factors to elucidate the underlying molecular mechanisms of BC. Genomics, transcriptomics and proteomics, along with single-cell analyses, have already been demonstrated to be effective for the discovery of new biomarkers and therapeutic targets. Advances in technology have enabled researchers to delve deeper into the molecular mechanisms of tumorigenesis, leading to the discovery of novel diagnostic and prognostic biomarkers and paving the way for the development of targeted therapeutic interventions. In the future, bioinformatics will be strongly related to clinical personalized medicine; by detecting patient-specific molecular profiles, personalized therapeutic agents may indeed be chosen in precision oncology, which could improve clinical results [13].

Herein, we will discuss applications of these bioinformatic strategies in changing our comprehension of BC, as well as how these are being employed to devise precision treatment approaches.

## 2. Bioinformatics Tools and Techniques for Biomarker Discovery

Bioinformatics is an interdisciplinary field that develops and applies mathematical, statistical, and computational methods to solve biological problems. It encompasses the utilization of software tools and databases for collection, organization, and analysis of large-scale biological and medical data, to gain a deeper understanding of biological systems. It mainly relies on “omics” technologies (genomics, transcriptomics, epigenomics, proteomics, lipidomics, metabolomics), each comprehensively focusing on a specific set of biological molecules.

### 2.1. Data Acquisition

**Figure 2:**
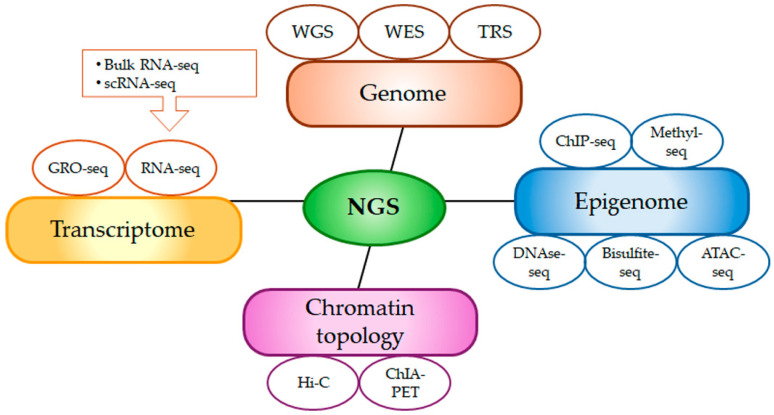
Overview of next-generation sequencing (NGS) applications in biomedical research. ATAC-seq: Assay for Transposase-Accessible Chromatin using sequencing; ChIA-PET: Chromatin Interaction Analysis by Paired-End Tag sequencing; ChIP-seq: Chromatin Immunoprecipitation sequencing; DNase-seq: DNase I hypersensitive sites sequencing; GRO-seq: Global Run-On sequencing; Hi-C: High-throughput Chromosome Conformation Capture; NGS: next-generation sequencing; RNA-seq: RNA sequencing; scRNA-seq: single-cell sequencing RNA TRS: Targeted Resequencing; WES: whole-exome sequencing; WGS: whole-genome sequencing; Methyl-seq: Methylation sequencing; bisulfite-seq: Bisulfite sequencing.

Modern sequencing techniques, often referred to as high-throughput sequencing or next-generation sequencing (NGS) (Figure 2), have transformed nucleic acid analysis by providing increased speed, lower costs, and higher accuracy compared to first-generation Sanger’s genome sequencing [14]. One NGS approach is whole-genome sequencing (WGS), which involves sequencing an organism’s entire genome using DNA extracted from blood or biopsy tissues [15]. WGS is highly sensitive to structural variants, including deletions, insertions, inversions, duplications, and translocations. Furthermore, it enables the identification of single-nucleotide polymorphisms (SNPs), which can contribute in elucidating the underlying biology of diseases and supports the development of effective, personalized treatment strategies [16,17]. Unlike WGS, which analyzes all genomic regions, the whole-exome sequencing (WES) focuses exclusively on the coding regions. Approximately 85% of mutations associated with diseases occur within the exons and WES covers around 95% of these areas, highlighting its significant role in research [18]. While both WGS and WES provide robust coverage of coding regions, WES may be more susceptible to sequence bias in large-scale applications, whereas WGS offers more reliable detection of structural variants [19]. Finally, Target Region Sequencing (TRS) analyzes a select set of genes or genomic regions with specific functions, often linked to particular diseases or phenotypes [20]. This technique has a wide range of applications, including detection of SNPs, insertions and deletions, copy number variations, and structural variants. It also allows discovery of germline or somatic mutations, linkage analysis for inherited diseases, and identification of biomarkers and therapeutic targets; one of the significant advantages is the TRS ability to identify variants at low allele frequencies, even as low as 1%.

RNA sequencing (RNA-seq) is the preferred technique for studying the transcriptome and serves as a primary tool for expression analysis of coding and non-coding (nc) RNAs [14]. Both bulk (which measures average gene expressions across large cell populations) and single-cell RNA (scRNA-seq, which analyses RNA profiles at the single-cell level) sequencing are developed for gene expression analysis [21]. These methods should be viewed as complementary rather than interchangeable [22]. Additionally, global run-on sequencing (GRO-Seq) allows mapping the production of active and nascent RNA, capturing transcription activity in real time rather than steady-state RNA levels [23]. These techniques can be useful for deciphering mechanisms of drug resistance occurring during treatment of several diseases, including cancer, and for developing personalized medicine approaches tailored to each patient’s molecular profile [17].

More recent NGS applications include those employed in epigenomics and chromosome conformation and interaction technologies. The first one commonly utilizes chromatin immunoprecipitation followed by sequencing (ChIP-seq) for mapping histone modifications [24], methyl-seq and bisulfite-seq (these techniques enzymatically or chemically convert unmethylated cytosines into uracil, later read as thymidine) for DNA methylation studies [25], ATAC-seq (a technique that creates tagged DNA fragments, using a hyperactive mutant Tn5 transposase, subsequently PCR-amplified and sequenced) and DNase-seq (it consists in sequencing of DNase I-sensitive DNA regions not occupied by histones) for studying chromatin accessibility [26]. Investigations on dynamic and regulated interplay of macromolecules within the cell utilize High-throughput Chromosome Conformation Capture (Hi-C) and Chromatin Interaction Analysis with Paired-End-Tag sequencing (ChIA-PET), the last combining ChIP and sequencing for mapping long-range chromatin interactions [27,28].

The main techniques used in proteomics involve gel, chromatography, reverse-phase microarrays, and mass-spectrometry-based analyses, together with bioinformatic approaches [29]. Proteins are firstly isolated from a mixture through 2D gel electrophoresis, affinity chromatography, size exclusion chromatography or ion-exchange chromatography. Protein identification is the next step, which makes use of several techniques, among which the most common is mass-spectrometry, where proteins are separated according to their mass-to-charge ratio (*m*/*z*), enabling their identification [29].

Finally, by using nuclear magnetic resonance (NMR), protein microarrays, and mass spectrometry, it is possible to investigate the complete set of low-molecular-weight metabolites (<1500 Da) produced by a cell because of its metabolic activity. In this context, cellular and organismal metabolic functions can be studied by considering both endometabolome (which consists of all intracellular metabolites) and exometabolome (which includes metabolites secreted into the growth medium or extracellular fluid) [30].

### 2.2. Data Collection and Organization

The holistic understanding of interactions and relationships within biological systems requires a systematic approach to data acquisition and organization. The advent of online databases with open access has enabled researchers to utilize, analyze, and interpret this wealth of information effectively [31].

Databases can be categorized into two main categories: primary and secondary databases [32]. Primary databases contain experimental data that researchers generate and submit directly. An example is represented by GenBank, a comprehensive genetic sequence database that houses nucleotide sequences primarily obtained through submissions from individual laboratories [33]; this database plays a crucial role in storing and disseminating publicly available DNA sequences. Secondary databases consist of curated analyses and interpretations of primary data, making them more refined [32].

Databases are further classified according to the specific type of biological data they curate (Table 1):

(1) Sequence databases. These encompass both primary and secondary collections of DNA, RNA, or protein sequences. Besides the above-mentioned GenBank collection, other online available databases include the following: (i) EMBL (EMBL Nucleotide Sequence Database), a comprehensive repository of nucleotide sequences and annotations maintained by EMBL’s European Bioinformatics Institute (EMBL-EBI), drawing data from public databases [34]; (ii) RNAcentral, a public resource that provides integrated access to a continually updated, extensive collection of non-coding RNA sequences [35]; (iii) UniProt, a freely accessible resource for protein sequences and their functional annotations. The UniProt Knowledgebase (UniProtKB), containing over 227 million sequences, is continuously updated by the UniProt team using machine learning and data extracted from scientific literature [36].
(2) Gene expression databases. These store information about gene expression patterns across various cell types, tissues, or organisms at different times or under specific conditions. These databases enable, for example, the comparison of gene expression levels in healthy versus tumor tissues or in tissues treated with a placebo versus those treated with a drug. Gene Expression Omnibus (GEO) is a public functional genomics data repository, enabling researchers to explore and analyze gene expression data, including both raw and processed data; it also offers web tools that allow users to analyze and interpret data [37]. The Cancer Genome Atlas (TCGA) is a comprehensive database, containing over 20,000 tumor and matched normal samples across 33 of the most prevalent forms of cancer, all molecularly characterized at the DNA (copy number changes, epigenetic modifications), RNA (messenger RNA and microRNA), and protein levels [38].
(3) Genetic databases. These contain information on genetic variants, including mutations, SNPs, and other genomic modifications linked to genetic diseases and pathological conditions. Examples include the following: (i) Clinical Genome Resource (ClinVar) catalogs various types of structural variants (SNPs, copy number variations, inversions, and translocations) together with their association with diseases [39]. Clinical relevance information for these variants is contributed by clinical testing labs, research institutions, and expert groups [39]. (ii) Single-Nucleotide Polymorphism Database (dbSNP) stores information on single nucleotide variants, microsatellites, insertions, and deletions that are prevalent in the human genome, useful for cancer research and genetic association studies [40,41].
(4) Molecular structure databases. These provide access to the three-dimensional (3D) structures of biological molecules. Understanding these structures is critical for elucidating their functions and roles within cells. Among the most significant databases deserve mention: (i) Protein Data Bank (PDB), an open-access repository that houses over 210,000 experimentally validated 3D structures of proteins and nucleic acids [42]. The database is weekly updated with relevant functional annotations sourced from various external biodata resources [43]; (ii) Structural Classification of Proteins (SCOP), a database that organizes proteins with known 3D structures based on their evolutionary and structural relationships [44].
(5) Molecular interaction databases. These focus on biomolecular interactions, particularly protein–protein interaction (PPIs). By identifying biological pathways, molecular patterns, and discovering new protein functions, these databases can elucidate the molecular basis of various pathologies, making them valuable tools for prevention, diagnosis, and therapy [45]. Databases in this category include the following: (i) Biological General Repository for Interaction Datasets (BioGRID), which provides comprehensive information on protein and genetic interactions across multiple species (including yeast, mice, and humans), thus allowing users to create intricate network graphs [46]; (ii) STRING, a key resource for studying physical and functional PPIs, deriving from experimental interaction databases, scientific literature, and computational predictions based on co-expression [47]; (iii) IntAct, a curated database system and analysis tool for investigating molecular interactions derived from scientific literature and direct data submissions. IntAct features over one million binary interactions and is continuously updated, with annotations that detail how even minor sequence changes can affect protein interactions [48].
(6) Biological Pathway Databases. These provide valuable insights into the biological roles of molecules and the metabolic pathways they participate in. The most common functional database is Kyoto Encyclopedia of Genes and Genomes (KEGG), a comprehensive database designed to assign functional meanings to genes and genomes at both molecular and broader biological levels. This integrated resource combines 15 manually curated databases with one computationally generated database, organized into four main categories (systems, genomic, information, and health information). KEGG serves as a vital tool for studying metabolism, genetic pathways, organismal functions, and human diseases [49].

**Table 1:** Major public databases for omics data integration and analysis in biomedical research.

Database	Name	Details	Website (10 June 2025)
Sequence	GenBank	DNA sequences	https://www.ncbi.nlm.nih.gov/genbank/
	EMBL	Nucleotide sequences and annotations	https://www.ebi.ac.uk/embl/
	RNAcentral	Non-coding RNA sequences and annotations	https://rnacentral.org/
	UniProt	Protein sequences and annotations	https://www.uniprot.org/
Gene expression	GEO	Multi-omics data	https://www.ncbi.nlm.nih.gov/geo/
	TCGA	Multi-omics data	https://www.cancer.gov/ccg/research/genome-sequencing/tcga
Genetic	ClinVar	Genetic variants and associations with diseases	https://www.ncbi.nlm.nih.gov/clinvar/
	dbSNP	Small genetic variations	https://www.ncbi.nlm.nih.gov/snp/
Molecular structure	PDB	3D structure of proteins, nucleic acids and complexes with functional annotations	https://www.rcsb.org/
	SCOP	Evolutionary structure and structural relationships of proteins	https://scop.mrc-lmb.cam.ac.uk/
Molecular interactions	BioGRID	Protein, genetic and chemical interactions	https://thebiogrid.org/
	STRING	Protein–protein interactions	https://string-db.org/
	IntAct	Molecular interactions for macromolecular complexes	https://www.ebi.ac.uk/intact/
Biological Pathways	KEGG	High-level functions of biological systems	https://www.kegg.jp/

BioGRID: Biological General Repository for Interaction Datasets; ClinVar: Clinical Variation; dbSNP: Single-Nucleotide Polymorphism Database; EMBL: European Molecular Biology Laboratory; GEO: Gene Expression Omnibus; KEGG: Kyoto Encyclopedia of Genes and Genomes; PDB: Protein Data Bank; RNAcentral: RNA Central Database; SCOP: Structural Classification of Proteins; STRING: Search Tool for the Retrieval of Interacting Genes/Proteins; TCGA: The Cancer Genome Atlas; UniProt: Universal Protein Resource.

### 2.3. Data Analysis

The final step in the bioinformatics pipeline is represented by data analysis, where raw data are converted into meaningful biological insights. It is the conclusion of all preceding steps (data acquisition, collection, and organization) and utilizes computational tools and algorithms to extract relevant information. Open-access tools, together with publicly available databases, empower researchers to collaborate and share data, thereby accelerating progress in molecular research.

While not exhaustive, here we describe a selection of the most common web-based tools employed in bioinformatics analyses, all of which are freely available and thus more accessible to the broader scientific community (Table 2).

At the end of NGS procedures many short read sequences are generated, and alignment is necessary to identify the corresponding segment of each sequenced read in its reference sequence [50]. Sequence alignment is fundamental in comparative studies and phylogenetics analysis, since it allows comparison among two or more sequences (DNA, RNA, peptides or proteins) to highlight similarities and differences, for understanding potential functional, structural or evolutionary relationships. Alignment is evaluated using a scoring system: for nucleotides, a positive score is given when identical bases are present in both sequences, while protein scoring considers chemical and physical features of amino acids (amino acids with similar characteristics receive higher scores) [50]. The most commonly used amino acid substitution scoring matrices include PAM [51], BLOSUM [52], and JTT [53].

Splicing variants from TCGA can be analyzed by Tumor Splicing Variants database (TSVdb) [54], a user-friendly interface for visualization of gene expression, splicing patterns and clinical data, thus allowing understanding of the relationships between isoforms and patient prognosis. Another online platform for the analysis of multidimensional cancer genomics is the cBioPortal for Cancer Genomics, which outputs complex molecular profiling data (deriving from cell lines or tumor tissue) in an easily interpretable format (containing genetic, epigenetic, gene expression, and proteomic information) [55].

Accurate discrimination of long non-coding (lnc) RNAs from protein-coding genes within transcriptomic data is achieved using LncRNA-ID, a powerful tool that employs a machine learning model to assess coding potential based on a comprehensive set of transcriptional features [56]. Another useful online tool is LncBook 2.0 that incorporates lncRNA annotations at different omics levels, thus allowing to decipher lncRNA signatures in diffrent physiopatological contexts [57].

User-friendly web platforms, such as miRNet 2.0 [58], miRTargetLink 2.0 [59], and miRDB [60], are also available for exploring miRNA-centric networks; they allow understanding of the intricate relationships between miRNAs and their target genes, as well as their role in several biological processes and diseases.

A major application of RNA-seq data is, however, the analysis of differentially expressed genes (DEGs), allowing researchers to compare gene expression levels across samples under different conditions. For example, RNA-seq can be employed to investigate gene expression variations between healthy and tumor tissues, providing insights into molecular mechanisms underlying disease and facilitating the identification of potential biomarkers or therapeutic targets [17]. The functional roles of DEGs can be inferred through Gene Ontology (GO) analysis, which establishes a hierarchical structure encompassing biological, cellular, and molecular functions associated with genes [61]. It is continuously updated to reflect new scientific discoveries, ensuring it remains current with the latest advancements in biological knowledge [62]. GO term annotation analysis (mapping the provided entries to GO subsets) or enrichment analysis (scanning for GO categories that are overrepresented in the input list) can be performed through the open-source web applications Gonet [63] and ShinyGO 0.82 [64]. Easy Visualization and Inference Toolbox for Transcriptome Analysis (eVITTA) is a powerful tool providing modules for analyzing and exploring studies published in NCBI GEO (easyGEO), detailed molecular- and systems-level functional profiling (easyGSEA), and customizable comparisons among experimental groups (easyVizR) [65]. Other commonly used web-based tools are Integrated Differential Expression and Pathway analysis (iDEP) 2.0 [66] and gene Profiler (g:Profiler) [67].

AlphaFold represents a breakthrough in protein structure prediction. It is an artificial intelligence (AI) program, developed by DeepMind and EMBL-EBI, that predicts the 3D structure of a protein based on its primary amino acid sequence, by using machine learning. This tool has transformed bioinformatics and molecular biology research fields, by enabling accurate prediction of protein structures, crucial for understanding protein functions and interactions with other biomolecules. The latest version, AlphaFold 3, released in 2024, can predict not only the structure of proteins but also that of DNA and RNA, as well as identifying ligands and their interactions. This advancement is made possible through a deep learning architecture, representing a significant leap forward in comprehending complex biochemical interactions [68].

Finally, molecular docking is a computational method used to predict binding affinity between ligands and receptors, particularly useful in drug discovery [69]. The process involves two main steps: (i) predicting the binding pose of the compound, which is the most energetically favorable arrangement when the compound is attached to the target, and (ii) estimating the binding energy that holds the compound to the target. Two key inputs are required: the 2D chemical structure of the compound and the 3D structure of the target, which can be obtained by X-ray crystallography or nuclear magnetic resonance spectroscopy [69,70,71]. AutoDock Vina 1.2.0 [72] and CB-Dock2 [73] are some of the freely accessible docking tools.

### 2.4. Machine Learning and AI

Biological data are challenging to analyze, because of their complexity; therefore, there is a growing use of machine learning and AI tools, aimed at creating informative and predictive models of the underlying biological processes [74]. These tools are especially valuable for handling datasets that are too large or intricate for human analysis, as well as for automating data analysis tasks. The fields of machine learning and AI are continuously evolving, as demonstrated by the sharp rise in global publication trends from 2018 to 2023, underscoring the growing influence of deep learning, especially BC diagnosis and treatment [75]. While they are not a one-size-fits-all solution particularly in cases where datasets are insufficient or when the focus is on understanding rather than prediction, they remain a crucial resource in biological research [74].

## 3. Bioinformatics in BC Research

Despite advances in BC research, mortality persists, and its rates continue to rise, highlighting the persistent difficulties in early detection, personalized treatment, and ongoing monitoring. Consequently, identifying novel biomarkers is crucial for improving patient outcomes, and multi-omics technologies and bioinformatics-driven integration of the resulting datasets are yielding clinical breakthroughs in personalized treatment and prognostics.

### 3.1. Diagnostic and Prognostic Biomarkers

Early detection represents a cornerstone of improved survival rates; nonetheless, currently it relies on imaging and histopathology, which may lack sensitivity for precancerous lesions or minimal residual disease, thus requiring novel diagnostic biomarkers. Novel biomarkers like gene expression signatures (multigene signatures), circulating tumor DNA (ctDNA) and ncRNAs offer non-invasive, high-resolution tools for earlier detection. This could revolutionize screening, particularly for high-risk groups, like those with TNBC, who remain difficult to deal with.

The combined analysis of two BC microarray datasets, globally encompassing 30 BC and 33 normal breast samples, identified a total of 733 DEGs; GO enrichment and KEGG pathway analysis, as well as construction of PPI network, lead to identification of 10 hub genes, strictly linked to growth and proliferation, among which six were strongly associated with BC progression. These DEGs are intimately linked to BC, displaying elevated expression across diverse BC subtypes, especially in TNBCs and being notably correlated with diminished patient survival rates [76] (Table 3).

Bioinformatics can also represent a powerful tool for classifying BC functional subtypes and stages, as well as for assessing the best treatment and predicting recurrence [96,97]. For instance, Li and coworkers [98] reported a computational framework combining transcriptomic profiling (101 normal breast and 218 TNBC tissue samples from GEO database) and protein interaction network to identify molecular drivers of TNBC. Fifty-four TNBC-related genes were identified, mostly related to invasion and metastasis and viral carcinogenesis. The authors also developed a novel high-risk BC prediction model, by implementing a pre-existing algorithm, whose accuracy reached 95.394% for TNBC diagnosis and 86.598% for TNBC staging.

By using the 80-gene molecular subtyping BluePrint test, Kuilman’s group identified and characterized a rare, but biologically distinct group of early-stage BCs, referred to as “dual subtypes” displaying both luminal basal and HER2 features [99].

Combination of WGS and RNA-seq approaches allowed identification of several fusion genes (ESR1-CCDC170, BCL2L14-ETV6, ETV6-NTRK3, MYB-NFIB, and NOTCH/MAST kinase) that may drive BC progression and may be useful for identifying patients who need closer monitoring and more aggressive therapy [100,101].

scRNA-seq performed on BC cells of axillary lymph nodes allowed a better knowledge of mechanisms underlying BC metastasis [79]. The authors analyzed five primary BC tissues (27,028 single cells) and 10 paired axillary lymph nodes (69,768 single cells), drawing a complete transcriptome profile visualized by the manifold learning and dimension reduction algorithm UMAP. The study allowed identification of nine cancer cell subclusters, including CD44^+^/ALDH2^+^/ALDH6A1^+^ BC stem cells, as well as key genes involved either in lymph node metastasis (*PTMA*, *STC2*, CST3, and *RAMP3* genes) or in interactions between metastatic cancer cells and immune cells (NECTIN2-TIGIT and LGALS1-PTPRC interactions). Based on these findings, the authors concluded that BC progression may be predicted evaluating the transcriptome profile, gene set score, and cellular composition of cancer cell clusters [79].

Recent classification systems leveraging protein expression profiling have been proposed to more effectively discern the functional phenotypic variations contributing to BC complexity and heterogeneity; a more precise classification, indeed, can guarantee enhanced prognostic accuracy and improved treatment outcomes. For instance, proteomic profiling of 300 formalin-fixed paraffin-embedded (FFPE) BC specimens revealed distinct protein signatures linked to different immune responses and clinical outcomes. In particular, basal-like BCs can be classified into two subgroups (immune hot/favorable prognosis and immune cold/unfavorable prognosis); HER2-enriched BCs can be distinguished in three subgroups, depending on lipid metabolism, immune-response and extracellular matrix characteristics, while in TNBCs, four proteomic clusters (basal-immune hot, basal-immune cold, mesenchymal, and luminal) can be recognized [84].

Likewise, Jeon and coworkers [85] conducted a proteomic analysis of 56 FFPE BC biopsies based on immune subtypes (immune-inflamed, immune-excluded, and immune-desert) to investigate the relationship between immune characteristics and clinical outcomes. Although no differences in prognosis were detected, the three groups displayed differences in terms of proteomic signatures. The immune-inflamed group, who responds better to immune checkpoint inhibitors, exhibited higher levels of coronin-1A, while immune-excluded/desert tumors (associated with an unfavorable response to immunotherapy) displayed upregulated α-1-antitrypsin levels. Furthermore, a positive correlation was observed between tumor-infiltrating lymphocytes (TILs), known to improve prognosis and treatment responses, and coronin-1A expression, while α-1-antitrypsin levels were negatively correlated with TILs [85].

Additionally, through high-throughput mass spectrometry, bioinformatic, and machine learning approaches, Azevedo and colleagues [86] have drawn protein expression patterns specific to each BC subtype. Notably, TNBCs exhibited the most extensive proteomic alterations, with 343 overexpressed and 121 downregulated proteins. Distinct biological pathways and molecular changes were linked to each BC subtype, alongside unique patterns of oncoprotein and tumor suppressor expression. Similarly, proteomic profiling of 60 human BC cells identified 13,000 proteins, enabling subtype classification. Importantly, specific protein signatures correlated with hormone receptor (ER, PR, HER2) status, underscoring their potential utility in biomarker discovery and drug development [89].

Metabolomic studies have further delineated pathways associated with BC onset and progression. In one comprehensive study analyzing both the metabolome and lipidome of 330 TNBC samples and 149 paired normal breast tissues, TNBCs were stratified into three distinct metabolomic subtypes: (i) C1-enriched in ceramides and fatty acids; (ii) C2-enriched in oxidation-related metabolites and glycosyl transfer products; (iii) C3-characterized by minimal metabolic dysregulation [82]. Integrating these data with available genomic and transcriptomic profiles revealed subtype-specific metabolites as potential therapeutic targets. For instance, the transcriptomic luminal androgen receptor subtype overlapped with C1, where sphingosine-1-phosphate (a key metabolite in the ceramide pathway) emerged as a promising therapeutic candidate; meanwhile, the transcriptomic basal-like immune-suppressed subtype corresponded with C2 and C3, where *N*-acetyl-aspartyl-glutamate emerged as another potential target [82]. Elia and coworkers [102] linked proline catabolism to in vivo metastasis formation, further demonstrating that inhibition of proline dehydrogenase was sufficient to impair lung metastasis development in mouse models.

Also, changes in the epigenomic landscape serve as key discriminators between healthy and cancerous breast tissues. We recently identified a critical feed-forward regulatory loop involving EBF1, ETS2, KLF2 transcription factors, and miR-126. Disruption of this EBF1/ETS2/KLF2-miR-126-gene circuit promotes oncogenic transformation and progression in BC. Compared to healthy cells, the three transcription factors were significantly downregulated in BC due to epigenetic silencing or a “poised but not transcribed” promoter state. This downregulation altered the expression of cancer-related genes, thereby facilitating malignant transformation [81].

Distinct epigenetic regulation patterns have been reported across BC subtypes. For example, Karsli-Ceppioglu and coworkers [103] observed aberrant gene regulation linked to changes in H3K9ac and/or H3K27me3 epi-marks, especially in TNBC, luminal B and HER2^+^ tumors. Among DEGs associated with these histone modifications (79 DEGs for H3K9ac and 37 genes for H3K27me3), key transcription factors such as *PAX3*, *DLX5*, *RUNX1*, and *GATA4* were strongly implicated in BC progression. Similarly, decreases in H3K9me2/3 levels coupled with increased activity of KDM3A/JMJD1A in BC contribute to aberrant expression of critical genes, including *MYC*, *PAX3*, *WNT5A*, and *CDKN2A/B*. These epigenetic alterations play pivotal roles during transformation and hold promise as diagnostic markers and therapeutic targets [104]. Beyond histone modifications, a wide array of epigenetic mechanisms—including DNA methylation and ncRNA regulation—drive essential processes underlying tumorigenesis and metastatic potential in BC. For a comprehensive overview of the BC epigenome, including these multifaceted alterations, see the detailed review in [105].

In 2023, Choi and Chae [87] introduced moBRCA-net, an interpretable deep learning-based framework for BC subtype classification utilizing multi-omics datasets: this framework consists of four modules (preprocessing, multi-omics data integration, omics-level feature importance learning, and classification) that, through a multi-omics integration strategy, allow combination of gene expression, microRNA expression, and DNA methylation information for BC classification. Similarly, by using the Multi-Omics Factor Analysis v2 (MOFA+), an advanced statistical framework integrating transcriptomic, proteomic, and metabolomic data [106], Sharma and coworkers [107] successfully distinguished highly aggressive BCs from less aggressive forms. Furthermore, by using the Cancer Integration via Multikernel LeaRning (CIMLR) algorithm, integrating genomic, methylation, transcriptomic, microRNA expression, and protein data, Malighetti and colleagues [108] identified three prognostic biomarkers (LMO1, PRAME, and RSPO2), whose overexpression was consistently associated with worst outcome in both primary and metastatic BC patients [108].

Finally, the application of omics technologies and bioinformatics to plasma analysis has emerged as a powerful approach in BC research, offering significant promise for early detection, molecular subtype differentiation, and therapy response prediction. This approach is minimally invasive, less time-consuming, and more cost-effective compared to traditional methods. Plasma metabolomics, empowered by advanced bioinformatics, is enabling the discovery of novel biomarkers and therapeutic targets. Plasma metabolome and proteinogram performed on 216 healthy, benign, and BC subjects identified specific metabolic profiles for each condition [83]. Specifically, glutamate and glutamine metabolism, as well as alanine, aspartate, and glutamate and arginine biosynthesis metabolism, were found to be downregulated in BC patients; moreover, among the 31 differentially expressed proteins, aspartate aminotransferase, L-lactate dehydrogenase B chain, glutathione synthetase, and glutathione peroxidase 3 were closely linked to these metabolic pathways [83]. Two recent studies further exemplify the prognostic and predictive power of this approach. The first study [88] analyzed associations between 2074 circulating proteins and the risk of nine common cancers in a cohort of 337,822 cancer cases. It identified 21 proteins associated with BC risk. Among them, nine proteins (AOC2, SPN1, CD160, RALB, GDI2, CPNE1, ULK3, CTSF, and PLAUR) showed colocalized associations with multiple BC molecular subtypes. Notably, PLAUR exhibited a strong positive association with overall BC risk and all subtypes except HER2-enriched tumors [88]. The second study [80] employed a rigorous two-phase analytical framework combining proteome/transcriptome-wide association studies with Mendelian Randomization to identify plasma proteins that are not only associated with, but also causally linked to, BC risk. This approach leveraged large-scale high-throughput datasets and included extensive validation and sensitivity analyses to ensure robustness. The study identified five plasma proteins with strong causal associations to BC: PEX14 and CTSF showed positive causal effects, while SNUPN, CSK, and PARK7 were negatively associated with risk. Importantly, PEX14 was the only protein with a strong causal effect specific to the ER^−^ BC subtype, underscoring its subtype-specific importance [80].

### 3.2. Harnessing Bioinformatics for BC Therapy

Beyond diagnosis, biomarkers are also indispensable for tailoring therapies to individual tumor biology [109], especially taking into the account the high intra- and inter-tumoral heterogeneity of BC that demands more precise risk stratification of patients based on their prognosis and avoiding overtreatment in low-risk cases. Over the past decade, indeed, targeted therapy has become a central focus in oncology research and clinical practice, fundamentally transforming the treatment landscape for many cancers, including BC. A pivotal role is played by chemogenomic profiling that, by integrating genetic data with drug sensitivity patterns, maps molecular drivers of treatment response, decodes resistance mechanisms, and optimizes treatment selection, particularly in aggressive TNBC subtypes. In this context, Savage and co-workers [110] developed a library, consisting of 37 patient-derived xenografts (PDX) from hard-to-treat BCs, which retain the molecular and phenotypic characteristics of the original patient tumors. By combining different technologies (WGS, RNA-seq, reverse-phase protein array, drug sensitivity testing), the authors have drawn a comprehensive molecular profiling and screening of metastatic potential and chemosensitivity of this PDX library, thus indicating that it can represent a valuable preclinical resource.

Pharmacogenomics also plays a pivotal role, as genomic testing can be used for identifying specific mutations or polymorphisms that drive cancer growth and/or influence responses to drug therapy. Through this approach, *BRCA1* and *BRCA2* mutations have been identified as targetable genetic mutations in metastatic BC; targeted therapies (usage of PARP inhibitors, such as olaparib) have indeed been shown to be effective in treating patients with BRCA-positive BCs [111].

By providing insights into BC molecular subtypes and risk of recurrence, the Prediction Analysis of Microarray 50 (PAM50) [112] appears useful for tailoring treatment plans. For instance, Ohara and collaborators [113] evaluated the response to neoadjuvant chemotherapy for ER^+^ BC, demonstrating that PAM50 gene expression profiling provided a more accurate prediction of response than immunohistochemistry alone [113].

Proteomic approaches have been used to predict treatment response as well. For instance, by analyzing a BC cohort of 113 FFPE samples, before and after chemotherapy, Shenoy and colleagues [90] identified two proteins involved in proline biosynthesis, PYCR1 and ALDH18A1, significantly associated with chemotherapy resistance in a subtype-specific manner. In this context, a high-throughput proteomic dataset of over 13,000 proteins from 60 human BC cell lines is publicly available that, when combined with other omics data (genomics, transcriptomics, phosphoproteomics), aids in identifying markers predictive of drug response according to cancer subtype [89].

A strong predictor of therapy response and outcome is represented by the characterization of the immune tumor microenvironment (ITME), a complex and heterogeneous network consisting of varying stromal and immune cell populations, extracellular matrix, and signaling molecules [114]. Several computational tools, such as digital cytometry, allow the study of ITME. For example, CIBERSORT, and its successor CIBERSORTx, uses bulk tumor RNA-seq data to digitally estimate the proportions of immune cell types, allowing a more affordable alternative to scRNA-seq [115]. To overcome bias linked to these methods (such as inaccurate quantification of tumor-infiltrating immune cells), Fernandez and coworkers [116] developed the MIXTURE algorithm deconvoluting cell-type proportions of bulk tumor samples by using a leukocyte-validated gene signature, thus improving the accuracy of immune cell proportions related to outcome and response to immune checkpoint blockade. Recently, Zerdes and colleagues [117] characterized the spatial organization of ITME in early BC patients through machine learning approaches: by using biopsies from patients enrolled in the EORTC 10994/BIG 1–00 randomized phase III neoadjuvant trial (NCT00017095), they suggested that machine learning algorithms shows promise in accurate characterization of the immune infiltrate, with valuable prognostic and therapeutic implications [117].

Although many drugs have been approved for BC treatment [118], new therapies are urgently needed to address the limitations of current options, including resistance, toxicity, and poor outcomes in aggressive or advanced cases. In recent years, combining biological and computational approaches (drug databases, tools for the analysis of drug–target interactions, molecular docking, and AI technologies) has offered promising strategies for drug repurposing [119,120,121]. Several drugs originally approved for non-cancer or other cancer indications are currently under active investigation, although formal FDA (Food and Drug Administration) approval for BC is still lacking for most of them (Table 4).

For example, integration of transcriptomic data and structural docking enabled to repurpose existing FDA-approved drugs, such as dolasetron and granisetron (two serotonin receptor antagonists, approved as anti-emetic in the context of cancer chemotherapy) that have been shown to behave as aromatase inhibitors, thus suggesting their usefulness in hormone-related BC treatment [94]. By interrogating the GSCALite web server, three potential repurposable drugs (the mitogen-activated protein kinase kinase inhibitors trametinib, selumetinib, and refametinib) were found to target key genes (*BUB1*, *ASPM*, *TTK*, *CCNA2*, *CENPF*, *RFC4*, and *CCNB1*) involved in BC development [92].

Several pre-clinical and clinical studies have underlined both direct (inhibition of mitochondrial activity and mTOR signaling, AMPK activation, with subsequent reduction in protein synthesis, proliferation and cell growth) and indirect (reduction in gluconeogenesis, inflammation, insulin and IGF-1 levels, and stimulation of glucose uptake) effects of metformin (an anti-diabetic drug) on BC [122]. In addition, by combining metabolic markers, dynamic FDG-PET-CT imaging, transcriptomics, and metabolomics in BC patients, two distinct metabolic adaptation patterns to metformin have been identified: the first one (linked to metformin-resistance) showed increased expression of oxidative phosphorylation and fatty acid oxidation genes, while the second one (linked to metformin-sensitivity) displayed increased glucose uptake [93,123].

Finally, a novel computational drug repurposing approach has been developed that integrates omics data into a network-based machine learning framework, effectively capturing the complex interactions among drugs, genes, and BC subtypes. Using this method, ruxolitinib was successfully identified as a potential new drug for personalized BC treatment [95].

**Table 4:** Examples of repurposed and investigational drugs for BC: original indications and current evidence.

Drug	Class	Original FDA-Approved Use	Repurposing for BC (Approval Status)	Refs
Anastrozole	Aromatase inhibitor	Therapy in postmenopausal women with advanced HR^+^ BC Adjuvant therapy in early HR^+^ BC	Postmenopausal women at high risk of developing BC (UK-approved)	[124]
Azelastine	Histamine receptor antagonist	Allergy	HR^+^, HER2^+^ and TNBC subtypes (not yet approved, pre-clinical study)	[125]
Diclofenac	COX inhibitor	NSAID for pain and inflammation	TNBC (not yet approved, pre-clinical study)	[126]
Metformin	Mitochondrial complex I inhibitor	Type-2 diabetes mellitus	HR^+^, HER2^+^ and TNBC subtypes (not yet approved, pre-clinical and clinical studies)	[122]
Nebivolol	β-adrenergic receptor antagonist	Hypertension	TNBC (not yet approved, pre-clinical studies)	[127,128]
Olaparib	PARP inhibitor	Advanced BRCA-mutated ovarian cancer	early and metastatic BRCA-mutated BC (FDA-approved)	[129]
Ruxolitinib	JAK inhibitor	Bone marrow and blood cancers	HR^+^ metastatic BC and TNBC (not yet approved, clinical study)	[130]
Trametinib	MEK inhibitor	BRAF-mutated melanoma, NSCLC, thyroid cancer, and low-grade gliomas	TNBC (not yet approved, clinical study)	[131]

BC: Breast Cancer; COX: Cyclooxygenase; FDA: Food and Drug Administration; HER2: Human Epidermal Growth Factor Receptor 2; HR: Hormone Receptor; JAK: Janus Kinase; MEK: Mitogen-Activated Protein Kinase Kinase; NSCLC: Non-Small Cell Lung Cancer; NSAID: Non-Steroidal Anti-Inflammatory Drug; PARP: Poly (ADP-Ribose) Polymerase; TNBC: Triple-Negative Breast Cancer; UK: United Kingdom.

In conclusion, integration of bioinformatics with genomic, proteomic, and pharmacological data is revolutionizing BC management by enabling increasingly precise therapy personalization. This multidisciplinary approach, indeed, not only allows understanding of the molecular mechanisms underlying therapy response but can also facilitate the discovery of novel biomarkers and/or the repurposing of existing drugs.

## 4. Conclusions

Due to the complex molecular landscape of BC, multi-omics approaches integrated with bioinformatics represent a relevant opportunity for more precisely defining the prognosis and selecting the best treatment for each individual patient. This field is continuously advancing, and some tests are already in use in some countries. Currently, five main gene expression profiling tests for BC are commercially available, namely Prosigna^®^ (PAM50), Mammaprint^®^ (based on the Amsterdam 70-gene BC gene signature), Oncotype DX^®^ (21-gene recurrence score assay), Breast Cancer Index^®^ (11-gene signature for predicting the risk of recurrence), and Endopredict^®^ (12 gene prognostic test) [132]. These tests provide valuable information to personalize therapies and improve disease management.

However, there are still many challenges that need to be overcome to make progress in this area. The first challenge is represented by the difficulty in integrating omics data, such as genomics, transcriptomics, and proteomics, to obtain a better understanding of biomolecular mechanisms, as each omics provides unique information about different aspects of the molecular system under study. Due to the dimensions and complexity of these data, sophisticated bioinformatics and expertise are required for analysis, where machine learning and AI play a crucial role by enhancing speed and precision.

AI supports knowledge-based approaches (such as identifying relationships between biomarkers through models that better integrate well-known biological structures) and manages challenges like noisy data, missing information from some omics technologies, and reducing reliance on manual feature engineering [133,134].

Another obstacle to address concerns big data generated by many sequencing methods. These data derive from different sources, are highly heterogeneous and, often, not related to each other. Moreover, the error rate is quite high, thus making it difficult to discriminate genetic variants from sequencing errors and to interpret data since many variants are not clinically relevant to the diseases [135]. These problems can be solved by combining data from different datasets, e.g., PharmGKB [136] and ClinVar [39], which curate and collate information on many variants, especially in the field of drug response [137].

The lack of published guidelines about bioinformatics may create confusion on how to establish and validate bioinformatics pipelines. Appropriate quality control is crucial to ensure that the generated data are robust, accurate, reproducible, and traceable. To address these challenges, the Association of Molecular Pathology issued clinical practice guidelines and reports (containing 17 consensus recommendations) designed to standardize the validation process of clinical NGS bioinformatics pipelines [138].

We mentioned above the role of machine learning and deep learning for diagnosis, staging, treatment, and prognosis in BC studies. However, AI challenges include nonuniformity of data standards, high development costs of AI systems, and instability of AI models. In particular, the latter problem is due to several factors, including incorrect data selection and accuracy, inherent bias, and model misspecification; thus, stability testing is mandatory for ensuring that AI works reliably. Going forward, employing multimodal learning to combine medical imaging with holographic data will become a valuable asset in research [139].

Looking ahead, bioinformatics will need standardized procedures and publicly accessible data for analysis to guarantee consistency and reproducibility. Increased investment in computational infrastructure and educational initiatives focused on bioinformatics and AI will further advance their use. To boost the reliability and broad applicability of AI-driven models, it is crucial to foster interdisciplinary collaboration and conduct comprehensive clinical validation [140].

## Figures and Tables

**Table 2:** Widely used databases and free web-based tools for bioinformatics analyses and related website links.

Database	Analysis	Website (10 June 2025)
AlphaFold 3	Prediction of protein, DNA and RNA structure and modeling of structural complexes	https://alphafold.ebi.ac.uk/
AutoDock Vina 1.2.0	Protein–ligand docking	https://vina.scripps.edu/
CB-Dock2	Protein–ligand blind docking	https://cadd.labshare.cn/cb-dock2/
cBioPortal for Cancer Genomics	Multi-omics cancer genomics data	http://cbioportal.org/
eVITTA	Transcriptome functional characterization	https://tau.cmmt.ubc.ca/eVITTA/
g:Profiler	Functional enrichment analysis at gene level	https://biit.cs.ut.ee/gprofiler/gost
GO	Gene functions, cellular processes and subcellular localization of proteins	http://www.geneontology.org/
GOnet	GO term annotation and enrichment analysis	https://tools.dice-database.org/GOnet/
iDEP 2.0	RNA-seq data analysis	https://bioinformatics.sdstate.edu/idep/
LncBook 2.0	human lncRNAs integration with multi-omics annotations	https://ngdc.cncb.ac.cn/lncbook/
LncRNA-ID	lncRNA identification	https://github.com/zhangy72/LncRNA-ID
miRNet 2.0	miRNA functions and interaction networks with genes, diseases, compounds, transcription factors.	https://www.mirnet.ca/
miRTargetLink 2.0	miRNA–mRNA interactions	https://ccb-compute.cs.uni-saarland.de/mirtargetlink2/
miRDB	miRNA–mRNA interactions and functional annotations	https://mirdb.org/mirdb/index.html
ShinyGO 0.82	Graphical gene-set enrichment	https://bioinformatics.sdstate.edu/go/
TSVdb	TCGA splicing variants	https://github.com/wenjie1991/TSVdb

CB-Dock2: Cavity Blind-Dock 2; eVITTA: easy Visualization and Inference Toolbox for Transcriptome Analysis; GO: Gene Ontology; g:Profiler: gene Profiler; iDEP 2.0: Integrated Differential Expression and Pathway analysis 2.0; lncRNA: long non-coding RNA; LncRNA-ID: Long Non-Coding RNA Identification; miRNA: microRNA; ShinyGO 0.82: Shiny Gene Ontology 0.82; TCGA: The Cancer Genome Atlas; TSVdb: Tumor Splicing Variants database.

**Table 3:** Most relevant studies on integrating experimental and bioinformatics strategies in BC research, conducted over the last five years (2020–2025).

BC Samples	Integrated Strategy	Main Findings	Refs
GEO and TCGA databases	Transcriptomic profiling PPI network construction Survival analysis	10 hub genes (*PBK*, *CCNA2*, *CDCA8*, *MELK*, *NUSAP1*, *BIRC5*, *CCNB2*, *HMMR*, *MAD2L1*, and *PRC1*) strongly associated with BC evolution.	[76]
GEO and TCGA databases	Transcriptomic profiling PPI network construction Survival analysis	23 hub genes negatively correlated with BC overall survival. Increased cell cycle gene (*CDK1*, *CDC20*, *AURKA* and *MCM4*) expression as predictive biomarker for poor prognosis.	[77]
TCGA and METABRIC databases	Transcriptomic profiling	Genes involved in cell communication (*CACNG4* and *CHRNA6*), cell cycle regulation and DNA replication *(PKMYT1*) pathways, and invasion and metastasis (*EPYC*) as diagnostic and prognostic markers.	[78]
Fresh tissues and axillary lymph nodes	Single cell transcriptomic profiling	CD44^ +^/ALDH2 ^+^/ALDH6A1^+^ cluster in BC stem cells. *PTMA*, *STC2*, *CST3*, and *RAMP3* genes involved in lymph node metastasis.	[79]
ARIC study	Transcriptomic and proteomic profiling	Five plasma proteins with strong and causal links to BC: PEX14 and CTSF positively associated; SNUPN, CSK, and PARK7 negatively associated.	[80]
Fresh tissues TCGA and GEO databases	Epigenomic and transcriptomic profiling PPI network construction	Identification of a TFs/miR-126/gene FFL regulating cell identity/stemness. FFL disruption promotes oncogenic transformation and BC progression.	[81]
Fresh tissues	Genomic, transcriptomic, metabolomic and lipidomic profiling. Gene–protein–reaction relationship construction	Subclassification of TNBCs in three metabolomic subtypes with prognostic value. *N*-acetyl-aspartyl-glutamate as potential therapeutic target for high-risk tumors.	[82]
Plasma samples	Metabolomic and proteomic profiling PPI network construction Machine learning models for diagnostic efficacy evaluation	Downregulation of metabolism of specific amino acids. Among the 31 DEPs, four enzymes (GOT1, LDHB, GSS, GPX3) linked to deregulated metabolic pathways. Identification of plasma metabolic signature for BC.	[83]
FFPE samples	Proteomic profiling Survival analysis	Subclassification of basal-like, HER2-enriched and TNBCs based on immune responses and clinical outcomes	[84]
FFPE samples TCGA database	Proteomic profiling Survival analysis	Coronin-1A and α-1-antitrypsin as markers for immune subtype-stratification	[85]
Fresh tissues NCG database	Proteomic profiling PPI network construction	Classification of BC subtypes based on oncoproteins/tumor suppressor DEPs.	[86]
TCGA database	Transcriptomic and epigenomic profiling	Development of a BC subtype classification framework (moBRCA-net).	[87]
Public protein-GWAS studies	Proteomic profiling/disease causal relationship construction	Genetically predicted concentrations of circulating AOC2, SPN1, CD160, RALB, GDI2, CPNE1, ULK3, CTSF, and PLAUR associated with BC risk and subtypes.	[88]
Cell lines	Proteomic profiling	~13,000 cell type-specific proteins correlated with HR status and molecular signatures. RB1 and CB2X as strong predictors of palbociclib response.	[89]
FFPE samples TCGA database Cell lines	Proteome and metabolome profiling PPI network construction Survival analysis	PYCR1 and ALDH18A1 associated with NAT resistance, tumor relapse and poor prognosis. *PYCR1* KO: increased glutamine catabolism and chemotherapy-sensitivity in ER^+^ cells, decreased integrin and laminin expression in ER^+^ and TNBC.	[90]
Cell lines Murine models	Transcriptomic profiling miRNA target gene prediction	EMT and metastasis inhibition by propranolol.	[91]
GEO and TCGA databases PDB and PubChem databases	Transcriptomic profiling PPI network construction Survival analysis TFs/miRNA/genes network construction Drug sensitivity analysis Molecular modeling and docking	Seven key genes (*BUB1*, *CCNB1*, *ASPM*, *TTK*, *CCNA2*, *CENPF*, and *RFC4*), regulated by specific TFs and miRNAs, involved in BC progression with prognostic value. Trametinib, selumetinib, and refametinib repurposing for BCs.	[92]
Fresh tissues	Transcriptomic and lipidomic profiling	Upregulation of fatty acid oxidation genes depending on metformin resistance or sensitivity.	[93]
CMap database	Transcriptomic profiling Molecular modeling and docking	Dolasetron and granisetron repurposing as aromatase inhibitors	[94]
METABRIC and LINCS databases	Genomic and transcriptomic profiling Drug–drug interaction analysis	Novel network-based approach for drug repurposing. BC subtype-specific ruxolitinib repurposing.	[95]

ALDH2: Aldehyde Dehydrogenase 2 Family Member; ALDH6A1: Aldehyde Dehydrogenase 6 Family Member A1; AOC2: Amine oxidase, copper containing 2; ARIC study: Atherosclerosis Risk in Communities study; ASPM: Assembly Factor for Spindle Microtubules; AURKA: Aurora kinase A; BC: Breast Cancer; BIRC5: Baculoviral IAP repeat containing 5; BUB1: Mitotic Checkpoint Serine/Threonine Kinase BUB1; CACNG4: Calcium voltage-gated channel auxiliary subunit gamma 4; CB2X: Cannabinoid receptor 2; CCNA2: Cyclin A2; CCNB1: Cyclin B1; CCNB2: Cyclin B2; CD160: CD160 antigen; CD44: CD44 Molecule (IN Blood Group); CDC20: Cell division cycle 20 homolog; CDCA8: Cell division cycle-associated 8; CDK1: Cyclin-dependent kinase 1; CENPF: Centromere Protein F; CHRNA6: Cholinergic receptor, nicotinic, alpha 6; Cmap database: Connectivity Map database; CPNE1: Copine-1; CSK: C-terminal Src kinase; CST3: Cystatin C; CTSF: Cathepsin F; DEPs: Differentially expressed proteins; EMT: Epithelial–Mesenchymal Transition; EPYC: Epiphycan; ER: Estrogen Receptor; ER2: Human Epidermal growth factor Receptor 2; FFL: Feed Forward Loop; FFPE: Formalin-Fixed Paraffin-Embedded; GDI2: GDP dissociation inhibitor 2; GEO: Gene Expression Omnibus; GOT1: Glutamic-oxaloacetic transaminase 1; GPX3: Glutathione peroxidase 3; GSS: Glutathione synthetase; GWAS: Genome-Wide Association Study; HMMR: Hyaluronan Mediated Motility Receptor; HR: Hormone Receptor; KO: Knockout; LDHB: Lactate dehydrogenase B; LINCS: Library of Integrated Network-based Cellular Signatures; MAD2L1: Mitotic Arrest Deficient-like 1; MCM4: Minichromosome Maintenance Complex Component 4; MELK: Maternal Embryonic Leucine Zipper Kinase; METABRIC: Molecular Taxonomy of Breast Cancer International Consortium; miR-126: microRNA-126; miRNA: microRNA; NAT: Neoadjuvant treatment; NCG: Network of Cancer Genes; NUSAP1: Nucleolar and Spindle Associated Protein 1; PARK7: Parkinsonism associated deglycase; PBK: PDZ Binding Kinase; PDB: Protein Data Bank; PEX14: Peroxisomal biogenesis factor 14; PKMYT1: Protein Kinase, Membrane Associated Tyrosine/Threonine 1; PLAUR: Plasminogen Activator, Urokinase Receptor; PPI: Protein–Protein Interaction; PR: Progesterone Receptor; PRC1: Protein Regulator Of Cytokinesis 1; PTMA: Prothymosin alpha; PYCR1: Pyrroline-5-carboxylate reductase 1; RALB: RAS-related protein Ral-B; Rb: Retinoblastoma 1; RFC4: Replication Factor C Subunit 4; SNUPN: Snurportin 1; SPN1: Spen family transcriptional repressor 1; STC2: Stanniocalcin 2; TCGA: The Cancer Genome Atlas; TF: Transcription Factor; TNBC: Triple-Negative Breast Cancer; TTK: Dual-specificity protein kinase TTK; ULK3: Unc-51 Like Kinase 3.

## Data Availability

Data sharing is not applicable to this article as no datasets were generated or analyzed during this current study.
